# The Anti-Atherosclerotic Effects of Endothelin Receptor Antagonist, Bosentan, in Combination with Atorvastatin—An Experimental Study

**DOI:** 10.3390/ijms25126614

**Published:** 2024-06-16

**Authors:** Marianna Stasinopoulou, Nikolaos Kostomitsopoulos, Nikolaos P. E. Kadoglou

**Affiliations:** 1 Center of Clinical, Experimental Surgery, and Translational Research, Biomedical Research Foundation, Academy of Athens, 115 27 Athens, Greece; mar.stasinopoulou@gmail.com (M.S.); nkostom@bioacademy.gr (N.K.); 2 Medical School, University of Cyprus, 2029 Nicosia, Cyprus

**Keywords:** endothelin receptor antagonist (ERA), bosentan, atorvastatin, atherosclerosis, plaque stability, matrix metalloproteinases

## Abstract

Bosentan, an endothelin receptor antagonist (ERA), has potential anti-atherosclerotic properties. We investigated the complementary effects of bosentan and atorvastatin on the progression and composition of the atherosclerotic lesions in diabetic mice. Forty-eight male *ApoE^−^/^−^* mice were fed high-fat diet (HFD) for 14 weeks. At week 8, diabetes was induced with streptozotocin, and mice were randomized into four groups: (1) control/COG: no intervention; (2) ΒOG: bosentan 100 mg/kg/day per os; (3) ATG: atorvastatin 20 mg/kg/day per os; and (4) BO + ATG: combined administration of bosentan and atorvastatin. The intra-plaque contents of collagen, elastin, monocyte chemoattractant protein-1 (MCP-1), tumor necrosis factor-a (TNF-a), matrix metalloproteinases (MMP-2, -3, -9), and TIMP-1 were determined. The percentage of lumen stenosis was significantly lower across all treated groups: BOG: 19.5 ± 2.2%, ATG: 12.8 ± 4.8%, and BO + ATG: 9.1 ± 2.7% compared to controls (24.6 ± 4.8%, *p* < 0.001). The administration of both atorvastatin and bosentan resulted in significantly higher collagen content and thicker fibrous cap versus COG (*p* < 0.01). All intervention groups showed lower relative intra-plaque concentrations of MCP-1, MMP-3, and MMP-9 and a higher TIMP-1concentration compared to COG (*p* < 0.001). Importantly, latter parameters presented lower levels when bosentan was combined with atorvastatin compared to COG (*p* < 0.05). Bosentan treatment in diabetic, atherosclerotic *ApoE^−^/^−^* mice delayed the atherosclerosis progression and enhanced plaques’ stability, showing modest but additive effects with atorvastatin, which are promising in atherosclerotic cardiovascular diseases.

## 1. Introduction

Τhe onset of endothelial dysfunction occurs prior to the formation of the atherosclerotic plaque. This process is multi-faceted, and the plaque core comprises various components of the innate immune system, such as macrophages, dendritic cells, and monocytes, along with elements of the adaptive immune system, including T cells [1,2]. Plaques that are prone to rupture are considered “vulnerable”, leading to the acute blockage of the artery and clinically to either acute ischemic heart attack or stroke [3,4]. The co-existence of diabetes mellitus (DM) multiplies the risk for acute plaque destabilization and consequent adverse cardiovascular events [5,6]. On the other hand, statins have been long proven to exert “pleiotropic” actions and remain an essential part of the secondary prevention of atherosclerotic cardiovascular diseases (ASCVDs). Among their beneficial impact, they stabilize vulnerable atherosclerotic plaques by inhibiting the inflammatory response and attenuating lipid deposition [7,8,9].

The activation of the endothelin (ET) system is closely linked to various pathological conditions, encompassing a broad spectrum of cardiovascular diseases, like essential hypertension, atherosclerosis, arterial restenosis, heart failure, coronary vasospasm, myocardial infarction, and pulmonary hypertension [10]. Endothelin was first described by Yanagisawa in 1988 and is known for its potent vasoconstrictor properties [11]. There are three isoforms of endothelin, namely, ET-1, ET-2, and ET-3, with ET-1 being the most abundant and relevant in the context of atherosclerosis [12]. ET-1 is a multisource derivative and exerts its effects by activating two distinct G protein-coupled receptors, ET_A_ and ET_B_. Both receptors are present on vascular smooth muscle cells (VSMCs), leading to vasoconstriction. Additionally, the ET_B_ receptor, which is also present on endothelial cells, mediates vasodilation by releasing nitric oxide (NO) or prostacyclin. ET-1 primarily acts in a paracrine manner, being secreted from endothelial cells towards the VSMCs of the arterial media [13]. In addition to myocardial and vascular fibrosis [14,15,16], ET further activates neutrophils, mast cells, and monocytes, influencing the production of a wide range of cytokines involved in the inflammatory cascade. Patients with diabetes typically exhibit elevated levels of ET-1 [17] and endothelial dysfunction, regardless of the presence of coronary artery disease (CAD) [18,19].

The pharmaceutical agent named bosentan acts as a dual endothelin receptor antagonist (ERA) against both ET_A_ and ET_B_. It has gained a significant place in pulmonary arterial hypertension (PAH), to relax pulmonary vasculature and inhibit the proliferation of VSMCs, promoting a favorable remodeling of pulmonary arterioles. Bosentan alleviates the symptoms associated with PAH and improves the overall condition of the patients [20,21]. In the case of ASCVDs, there is a small number of studies indicating the angio-protective effect on and the clinical improvement in patients with peripheral arterial disease [22]. Most importantly, it has been associated with less cardiovascular events [23]. Regarding mechanistic explanation, up to now, there is a single experimental study documenting the potential anti-atherosclerotic effect of bosentan, using a animal model of atherosclerosis (*ApoE^−^/^−^* mice) similar to the one used in our study. The study mainly focused on the effect of bosentan on protein and miRNA-21 and programmed cell death 4 (PDCD4) mRNA involved in the malfunction of endothelial cells, including the processes of inflammation and apoptosis. Based on their findings, it was hypothesized that bosentan may be an effective treatment for patients with atherosclerosis, as *ApoE^−^/^−^* mice had significantly smaller atherosclerosis plaques in vivo. This effect is likely due to the miRNA-21-mediated upregulation of the antiapoptotic protein Bcl-2 and the downregulation of PDCD4 and downstream proapoptotic effectors, caspase-3 and Bax, resulting in an overall antiapoptotic outcome [24]. Compared to that study, ours has focused on diabetic atherosclerosis which is usually aggressive and requires more intensive therapy. Moreover, we used a higher number of parameters to assess plaque stability in parallel to inflammatory factors contributing to plaque vulnerability for mechanistic investigation. Nevertheless, the actions of bosentan on arterial wall function and atherosclerosis unambiguously require more robust evidence [25].

In the present study, we aimed to evaluate the impact of bosentan on atherosclerotic plaque progression and vulnerability using a widely accepted animal model of diabetic atherosclerosis. Based on the well-known atheroprotective effects of statins, we explored the potential complementary action of bosentan to that of atorvastatin on atherosclerosis progression.

## 2. Results

### 2.1. Body Weight and Biochemical Analysis

Between groups comparison revealed no statistically significant difference in weight at the end of the study (*p* > 0.05). Similarly, there were non-significant differences in total triglycerides and glucose levels between all groups (*p* > 0.05). On the other hand, total cholesterol levels differed between groups at the end of the study. As expected, atorvastatin-treated mice (both ATG and BO + ATG) exhibited lower total cholesterol levels compared to controls (*p* < 0.05), whereas BOG and COG showed equivalent levels of total cholesterol at the end of the study (758 ± 182 mg/dL vs. 759 ± 131 mg/dL; *p* > 0.05) (Table 1).

### 2.2. Morphometry, Collagen, and Elastin

**Figure 1:**
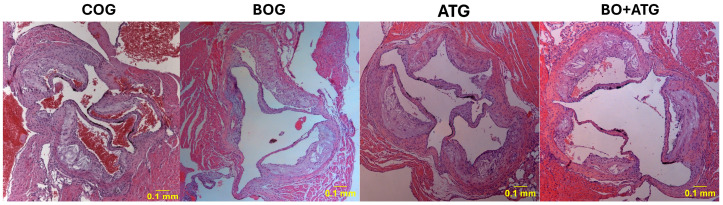
All active groups (BOG, ATG, and BO + ATG) had significantly smaller plaques compared to controls in *ApoE^−^/^−^* mice. Representative images and quantifications of aortic valve sections stained with hematoxylin/eosin across all groups.

The effect of bosentan treatment on atherosclerotic lesion burden, alone or combined with atorvastatin, was evaluated by measuring luminal stenosis at the level of aortic valve. The degree of luminal stenosis was significantly less in all treatment groups in comparison to COG (*p*< 0.001). Although the effect of bosentan administration on atherosclerosis was much less profound than that of atorvastatin, stenosis percentage was more limited in BO + ATG than in the group receiving atorvastatin alone (*p* = 0.045). Measurements are depicted in Table 2, and Figure 1 includes the representative images of the hematoxylin/eosin (H&E) staining.

Plaque stability was assessed by measuring collagen and elastin concentrations within the atherosclerotic plaques and fibrous cap thickness (Table 2). Compared to COG, sirius red staining revealed a statistically significant and higher collagen content along intervention groups: BOG (*p* = 0.023), ATG (*p* < 0.001), and BO + ATG (*p* < 0.001). Notably, atorvastatin yielded even greater increase in collagen content than bosentan (*p* < 0.01). A similar pattern was observed for the fibrous cap, where all interventions resulted in significant cap thickening than controls, and the atorvastatin-treated groups had even thicker fibrous cap over the bosentan-treated mice (ATG or BO + ATG vs. BOG; *p* < 0.05). In the case of elastin, atorvastatin treatment had the determinant role. In particular, bosentan did not significantly affect elastin levels compared to controls (*p* > 0.05). On the other hand, ATG and BO + ATG had considerably higher elastin levels than both BOG and COG (*p* < 0.001), implying that in the combined treatment atorvastatin administration drove the increase in elastin content. All morphometric data are presented in Table 2.

### 2.3. Immunohistochemistry

In comparison to COG, all treated groups showed higher a-actin concentrations, but only BOG and BO + ATG achieved statistically significant differences compared to controls (*p* < 0.05). The positive-stained area for macrophages was found smaller along BOG (*p* = 0.015), ATG (*p* < 0.001), and BO + ATG (*p* < 0.001) groups compared to COG, respectively. Atorvastatin was more effective than bosentan to reduce macrophage contents within plaques. Overall, bosentan and atorvastatin showed additive effects on the reduction in macrophage concentration (Table 2).

**Figure 2:**
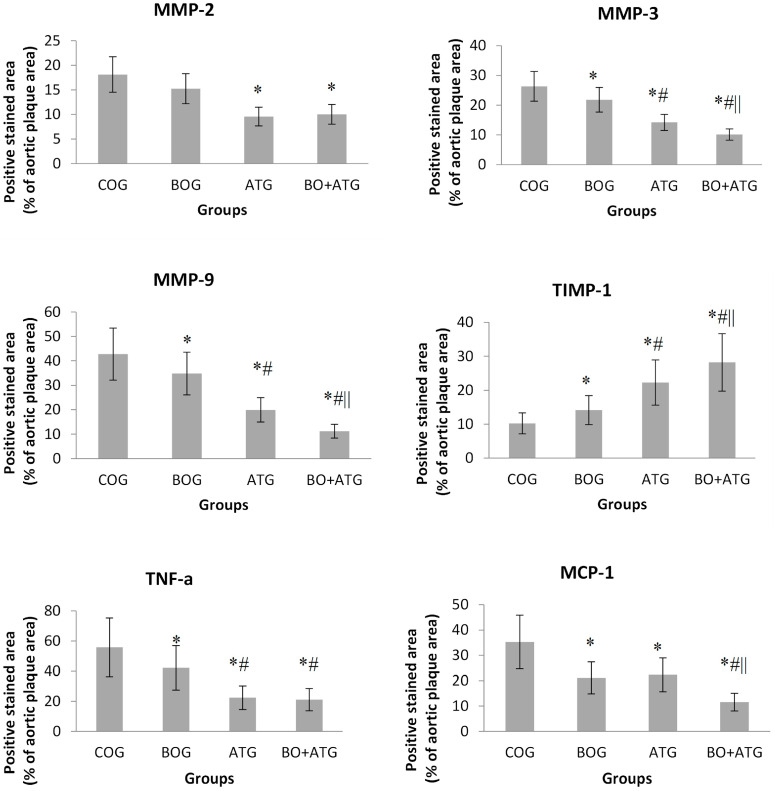
Quantification of immunohistochemical staining with antibodies against MMP-2, MMP-3, MMP-9, TIMP-1, TNF-a, and MCP-1. * *p* < 0.05 vs. COG, # *p* < 0.05 vs. BOG, || *p* < 0.05 vs. AΤG.

Atorvastatin treatment led to a considerable decrease in MMP-2 concentrations compared to COG (ATG: 9.56 ± 1.21%, BO + ATG: 10.02 ± 2.17% vs. COG: 18.12 ± 4.62%, *p* < 0.001). Given that the reduction in MMP-2 after bosentan treatment was not significantly different from that of the controls (15.24 ± 3.54%, *p* = 0.212), it appears that atorvastatin was solely responsible for the effect on MMP-2 in the group receiving the combined treatment (Figure 2).

All treatment groups were associated with statistically significant lower concentrations of MMP-3 and MMP-9, while TIMP-1 was statistically higher compared to COG (*p* < 0.05) (Figure 2). Bosentan showed complementary effects to atorvastatin in the above parameters, since its impact was significant but to a lesser extent than atorvastatin (*p* < 0.05), while the changes after combined treatment were greater than each single therapy with either bosentan or atorvastatin.

**Figure 3:**
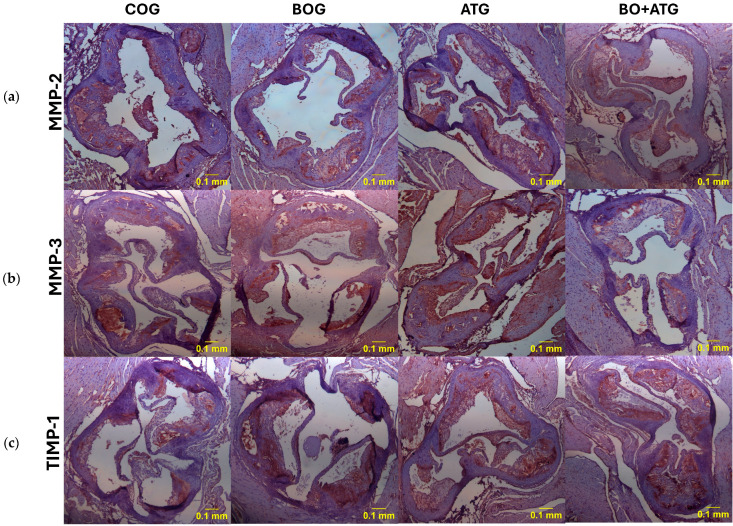
Representative images of immunohistochemical staining across all groups with antibodies against (**a**) MMP-2 (upper panel), (**b**) MMP-3 (middle panel), and (**c**) TIMP-1 (lower panel). Sections were counterstained with H&E.

Finally, the pro-inflammatory agents, TNF-a and MCP-1, presented a similar trend, with all interventions leading to significantly lower concentrations of these markers compared to COG (*p* < 0.05). After a single therapy of either bosentan or atorvastatin, TNF-a contents were considerably lower compared to those in controls (*p* = 0.023 and *p* < 0.001, respectively), with atorvastatin to a greater extent explaining the absence of significant difference between BO + ATG and ATG. On the other hand, MCP-1 concentrations were equivalently lower after single therapies of both bosentan and atorvastatin than control mice. Notably, their combination had complementary effects with further significant lowering of MCP-1 levels than all other groups (*p* < 0.05) (see Figure 2 and Figure 3).

## 3. Discussion

To our knowledge, this is the first experimental study demonstrating the beneficial effects of bosentan on diabetic atherosclerosis progression and vulnerability in a valid animal model. Moreover, this is the first time that an ERA has been combined with the established anti-atherosclerotic treatment of atorvastatin. Although lumen stenosis, fibrous cap thickening, and the increase in collagen content within plaque was modest after bosentan administration, it achieved statistically significant level compared to the untreated mice. Bosentan significantly ameliorated MMP-3, MMP-9, TIMP-1, TNF-a, and MCP-1 concentrations. Moreover, the addition of bosentan to atorvastatin administration showed a further significant lowering of the intra-plaque levels of MMP-3, MMP-9, TIMP-1, and MCP-1, implying the complementarity with statins and the favorable impact of bosentan on intra-plaque inflammation and stability (*p* < 0.05).

ET-1 represents the predominant isoform of endothelin, and it exerts paracrine and autocrine roles and is associated with deleterious effects in the cardiovascular system [26]. It triggers vasoconstriction through the activation of ET_A_ receptors and has been involved in the development of atherosclerosis. Both ET-1 and its receptors appear to be overexpressed in atheromatous lesions found in animal models as well as in humans [27]. In animals fed a HFD, ET-1 and its receptors have been also located in endothelial cells, VSMCs, and macrophages along different stages of atherosclerotic plaque progression [28,29]. A recent study using a valid animal model of atherosclerosis (*ApoE^−^/^−^* mice fed HFD) demonstrated lower atherosclerosis burden after the administration of an identical dose of bosentan [24]. Compared to that study, we confirmed the amelioration of atherosclerotic lesions in our animal model of diabetic atherosclerosis. Moreover, we comparatively evaluated the anti-atherosclerotic effect of bosentan and a statin member. The former had a more modest effect than atorvastatin in most examined parameters, which seemed unrelated to lipid homeostasis, since bosentan therapy left blood lipids unaltered in our study. The interplay of ET-1 with hyperlipidemia has been mentioned in patients with dyslipidemia and higher levels of ET-1 [30]. However, there is no clear evidence supporting the hypolipidemic effects of ERA [31]. Both lipid-lowering and “pleiotropic” actions of statins have been associated with atherosclerotic plaque stabilization in animal studies [32]. The positive effect of bosentan on atherosclerosis progression, beyond lipid modification, is resounding, but the extrapolation in clinical ASCVDs needs a further meticulous investigation.

In addition to atherosclerosis burden, we also assessed the complementary effects of bosentan to atorvastatin on atherosclerosis composition in *ApoE^−^/^−^* mice with diabetic atherosclerosis. We confirmed that, after 6 weeks of atorvastatin administration, all connective tissue components of plaque stability were improved (collagen, elastin and fibrous cap thickness). To our knowledge, this is the first study demonstrating plaque stabilization after bosentan administration either as a single therapy or on the top of atorvastatin therapy. We concluded that bosentan contributed to a more resilient plaque structure based on collagen, fibrous cap thickness, and VSMC content measurements. However, that effect was significantly smaller compared to the exaggerated amelioration of the same parameters that culminated in plaque vulnerability in atorvastatin receivers. Nevertheless, the beneficial effect of bosentan on plaque stability in an additive manner may encourage its use not as a stand-alone therapy but as a complementary regimen to established anti-atherosclerotic therapies, like statins. The use of pharmaceutical combinations has become a popular therapeutic approach in the clinical practice of ASCVDs, since it could inhibit a wider spectrum of atherosclerotic mechanisms and magnify their effectiveness [33,34]. This drug is currently available in clinical practice, but its use could be broadened if these effects on plaque stability were the same in people as those in mice [35].

MMPs and its inhibitor (TIMP-1) regulate the extracellular matrix (ECM) remodeling and inflammatory cell infiltration, determining atherosclerosis progression in diabetic condition [36]. The balance of proteolytic and anti-proteolytic enzymes (MMPs/TIMPs) has been long studied as a surrogate marker of plaque vulnerability [37]. To our knowledge, this is the first study demonstrating the favorable changes in MMP members, like MMP-3, MMP-9, and their inhibitor TIMP-1 within atherosclerotic lesions after bosentan therapy. Most importantly, the combined therapy with atorvastatin exerted a complementary effect of those two medications on MMPs/TIMP-1 homeostasis. In particular, the addition of bosentan to atorvastatin-treated mice further ameliorated intra-plaques contents of MMP-3, MMP-9, and TIMP-1, and those changes paralleled the beneficial changes in collagen and fibrous cap thickness after combined therapy. Perhaps, the downregulation of the proteolytic activity of MMPs/TIMP-1 in an additive pattern led to remarkably less degradation of collagen and elastin, enhancing the connective tissue scaffold within the atherosclerotic plaques. In addition to this, bosentan administration rather than atorvastatin triggered VSMCs’ accumulation in the plaques, the predominant cellular sources of the ECM [38]. Our findings are of clinical relevance, since the alteration in MMP/TIMP balance has been associated with atherosclerotic plaque vulnerability and adverse cardiovascular events in a clinical setting [39,40]. Hence, bosentan seems to possess the potential of reducing plaque vulnerability, even at a lower extent than atorvastatin, by shifting the balance between the production and degradation of ECM towards higher intra-plaque collagen and elastin within the atherosclerotic lesions [41]. The proposed combination could be clinically implemented concerning statins as an integral part of ASCVDs and bosentan as a novel add-on therapy.

A previous study supported the anti-atherosclerotic effect of bosentan, demonstrating its association with the suppressed expression of pro-apoptotic agents and increased miRNA-21 expression in the aortic arch endothelium [24]. Our study provides a novel anti-atherosclerotic mechanism of bosentan regarding its anti-inflammatory effects. We and other investigators have demonstrated the strong contribution of inflammation to atherosclerosis vulnerability [8,42,43]. We also noticed a complementary pattern after bosentan and atorvastatin in the modulation of inflammatory mediators, focusing on TNF-a and MCP-1. Experimental and clinical data have supported the relationship of inflammation with atherosclerosis development and progression [44]. Previous studies have reported the reduction in two multifunctional inflammatory agents, TNF-a and MCP-1, after statin administration [45,46]. A number of studies from a wide spectrum of ASCVDs have implicated the interaction between ET-1 and inflammation [47]. An increased expression of ET-1 receptors has been found in inflammatory cells (macrophages, T lymphocytes) and in the smooth muscle fibers of the vessels. It has been suggested that foam cells and T-lymphocytes regulate a switch in expression from ET_A_ to ET_B_ receptors in vascular endothelial smooth muscle fibers [48]. However, only in vitro data have reported that bosentan has the potential of suppressing inflammation through the modification of inflammatory agents like TNF-a and MCP-1 in models simulating atherosclerosis [49,50]. In our study, single therapy with either bosentan or atorvastatin inhibited macrophage infiltration and paralleled the lower macrophage-derived atherogenic cytokines, TNF-a and MCP-1, within plaques. The combination therapy seemed to precipitate further the suppression of TNF-a and MCP-1 and the consequent plaque stabilization with the highest elastin, collagen, and VSMC contents and the lowest macrophage concentration among groups. Many pharmaceutical interventions have targeted plaque stabilization, but the promising supplementary clinical relevance of bosentan remains to be proven.

The present study had several limitations. Firstly, we used histochemistry-based measurements that provide a semi-quantified molecular concentration but do not depict the absolute protein activity and its gene expression. Secondly, *ApoE^−^/^−^* mice fed HFD is a valid animal model of atherosclerosis, but plaque rupture is rarely identified, and thus, the estimation of plaque vulnerability is indirect. For this purpose, we and other investigators used parameters associated with plaque composition [51]. Thirdly, we could not examine the interaction between bosentan and atorvastatin. We limited our study to common parameters. Finally, we did not measure ET-1 levels and their receptors within plaques that could directly depict the impact of bosentan on ET-1 homeostasis within plaques.

In conclusion, the combined treatment of bosentan and atorvastatin in diabetic atherosclerotic *ApoE^−^/^−^* mice induced additive beneficial effects on plaque composition, favoring plaque stabilization. The amelioration of ECM content within plaques yielded a more stable plaque phenotype, which was associated with a beneficial modification of MMP-3, -9, TIMP-1 and MCP-1. After comparative evaluation of those pharmaceutical agents, bosentan did not show as much improvement in atherosclerosis parameters (burden and composition) as AT group, and its overall impact is considered modest. Our findings outline the potential clinical importance of pharmaceutical combinations in the management of established atherosclerosis. More studies are required to clarify the anti-atherosclerotic effects of bosentan as a supplement of other established therapies.

## 4. Materials and Methods

### 4.1. Animal Model and Experimental Design

All experiments were performed in the Biomedical Research Foundation of the Academy of Athens (BRFAA), and the protocol was evaluated and approved by the Veterinary Service of the Prefecture of Athens (permit number: 2697/26-04-2013), as required by the Greek legal requirements for animal experimentation. The facility in BFRAA is registered as a “breeding” and “experimental” facility according to the Greek Presidential Decree 56/2013, which harmonizes national legislation with the European Community Directive 63/2010 on the Protection of Animals Used for Scientific Purposes.

Male C57BL/6J ApoE double-knockout (*ApoE^−^/^−^* KO) mice, 8 weeks old, were used for the purposes of this study. All mice had ad libitum access to filtered tap water in drinking bottles and were fed a Western-type high-fat diet (HFD—Harlan, Teklad; 88137) for 14 weeks, in order to develop atherosclerotic lesions. Diabetes was induced after 8 weeks from the beginning of the study by intra-peritoneal injections of streptozotocin (STZ) for 5 consecutive days (0.05 mg/g body weight in 0.05 mol/L citrate buffer, pH 4.5). After STZ injections, mice maintaining fasting glucose levels of > 200 mg/dL were considered diabetic and served as models. Random glucose tests during the study period consistently confirmed the diabetic status of mice. We used a valid animal model for diabetic atherosclerosis development, which we have previously used in pharmaceutical studies [52,53]. Diabetic mice continued HFD and were equally divided into the following four groups (*n* = 12) for the last 6 weeks of the study period:

(1) Control group (COG): normal saline was administered every day by esophageal gavage to make all interventions comparable between groups.
(2) Bosentan group (BOG): bosentan (Actelion Pharmaceuticals LTD, Allschwil, Switzerland) was administered by esophageal gavage (100 mg/kg/day).
(3) Atorvastatin group (ATG): mice were treated with atorvastatin (20 mg/kg/day) that was administered by esophageal gavage. The detailed protocol has been described in previous publication [8].
(4) Bosentan and atorvastatin (BO + ATG): concomitant bosentan and atorvastatin administration by esophageal gavage, as described previously, for 6 weeks.

On the last day of the experimental period (14th week of experiments), body weights were measured after overnight fasting. All mice were euthanized by exsanguinations after the induction of anesthesia with isoflurane 5% (IsoFlo; Abbott, IL, USA). Whole blood samples were obtained at the time of euthanasia via cardiac puncture. These were initially centrifuged at 3000 rpm for 15 min and then immediately assayed in an automatic enzymatic analyzer (Olympus AU560, Hamburg, Germany). Parameters measured were fasting glucose, triglycerides, and total cholesterol plasma levels

### 4.2. Histological Parameters

After euthanasia, the heart along with the aortic root was perfused with normal saline via heart puncture. Afterwards, the heart along the aorta was excised and fixed in 10% buffered formalin overnight and then embedded in paraffin blocks. The procedure of aortic valve sectioning with microtome and paraffin blockage for histomorphometric analysis is valid and has been previously as described in [8]. We used three nonconsecutive aortic slices (at equal intervals of 20 μm) for each staining per mouse. Aortic plaque area and collagen and elastin contents were quantitatively assessed, by staining cross-sections with hematoxylin/eosin (H&E), sirius red, and orcein, respectively, according to a standardized protocol [49]. Image analysis software (Image Pro Plus version 4.1; Media Cybernetics; Rockville, MD, USA) was used for morphometric image analysis.

The paraffin sections of the aortic arch were stained immunohistochemically with antibodies directed against the Mac-3 antigen of murine macrophages (dilution 1: 50; BD Pharmingen, Franklin Lakes, NJ, USA) and the alpha-smooth muscle isoform of actin (dilution 1: 100; Biocare Medical, LLC, Concord, CA, USA). Moreover, MMP-2 and MMP-3 were identified with the corresponding polyclonal antibodies (dilution 1: 300; MBL, International Corporation, Woburn, MA, USA). A polyclonal antibody against mouse MMP-9 was purchased from AbD Serotec (Bio-Rad AbD Serotec Ltd, Kidlington, Oxfordshire, UK). Paraffin-embedded sections were examined immunohistochemically with the following antibodies: anti-mouse monoclonal antibody against TIMP-1 (dilution 1: 300; Acris Antibodies GmbH, Herford, Germany) and anti-mouse polyclonal antibody against MCP1 (dilution 1: 300; Acris Antibodies GmbH, Hiddenhausen, Germany). AEC staining kit with universal secondary antibody (Zytomed Systems GmbH, Berlin, Germany) and Avidin/Biotin Blocking Kit (Vector Laboratories, Burlingham, CA, USA) were also used. Sections were finally counterstained with hematoxylin. 

### 4.3. Histomorphometry

Using a Leica DM LS2bright-field microscope (Leica Microsystems, Wetzlar, Germany), we observed all sections from the aortic valves, and digital pictures were acquired using nd stored in a lossless format using high resolution CMOS color camera (ALTRA20, Olympus Soft Imaging Solutions GmbH, Münster, Germany) Total plaque area and total lumen area (in μm^2^) circumscribed by the internal elastic lamina (IEL) were measured in H&E-stained sections. The percentage of luminal stenosis was calculated as the proportion of the total lumen area occupied by all atherosclerotic plaques in each section, and then, we averaged luminal stenoses.

In sirius red- and orcein-stained sections, we measured the relative concentrations of collagen and elastin, respectively, and the fibrous cap thickness of each atherosclerotic plaque. For each parameter, we then averaged all values of all sections per mouse. For the measurement of the relative concentrations of the stained molecules by immunohistochemistry, the segmental stained plaque area was expressed as the percentage of the whole atherosclerotic plaque area [39]. We then averaged the values of all plaques per animal and then for each group.

### 4.4. Statistical Analysis

The results of the study are presented as mean values ± standard deviation. The normality of distribution was assessed using the Kolmogorov–Smirnov test. For continuous variables, the comparisons between groups were made using one-way ANOVA and post hoc Tukey test. We did not use non-parametric analysis due to the normal distribution of variables. A two-tailed *p* value of <0.05 was considered statistically significant. All statistical analyses were based on SPSS v26.0 (IBM, Armonk, NY, USA).

## Figures and Tables

**Table 1:** Body weight, lipid parameters, and glucose at the end of the study.

	COG (*n* = 12)	BOG (*n* = 12)	ATG (*n* = 12)	BO + ATG (*n* = 12)	*p*
Weight (g)	34.4 ± 4.2	33.3 ± 4.8	32.9 ± 3.9	33.5 ± 4.2	0.512
FPG (mg/dL)	281 ± 29	289 ± 55	295 ± 45	309 ± 49	0.794
TC (mg/dL)	759 ± 131	758 ± 182	511 ± 131 *	502 ± 191 *	<0.001
TG (mg/dl)	155 ± 41	145 ± 34	129 ± 30	131 ± 33	0.400

COG, control group; BOG, bosentan group; ATG, atorvastatin group; BO + ATG, bosentan + atorvastatin group. FPG, fasting plasma glucose; TC, total cholesterol; TG, triglycerides; and *p*, one-way ANOVA. * Tukey test, *p* < 0.05, vs. COG.

**Table 2:** Atherosclerotic lumen stenosis (percentage of plaques area/lumen area) (H&E staining), intra-plaque contents of collagen (sirius red staining), elastin (orcein staining), vascular smooth muscle cells (VSMCs—a-actin), and macrophages (Mac-3).

	COG (*n* = 12)	BOG (*n* = 12)	ATG (*n* = 12)	BO + ATG (*n* = 12)	*p*
Lumen stenosis (%)	24.6 ± 4.8	19.5 ± 2.2 ^a,c,d^	12.8 ± 4.8 ^a,b,d^	9.1 ± 2.7 ^a,b,c^	<0.001
Elastin (%) plaque	8.12 ± 2.10	10.62 ± 6.52 ^c,d^	25.17 ± 6.91 ^a^	31.02 ± 5.23 ^a,b^	<0.001
Collagen (%) plaque	14.21 ± 4.21	22.83 ± 4.79 ^a,c,d^	31.88 ± 5.97 ^a,b^	40.33 ± 8.72 ^a,b,c^	<0.001
Fibrous cap thickness (μm)	9.12 ± 3.10	13.12 ± 3.23 ^a,c,d^	23.12 ± 5.44 ^a,b,d^	48.12 ± 6.21 ^a,b,c^	<0.001
a-actin (VSMCs) (%) plaque	17.13 ± 3.21	26.88 ± 6.06 ^a^	20.53 ± 6.97	28.10 ± 6.82 ^a^	0.005
Mac-3 (macrophages) (%) plaque	34.56 ± 10.25	26.46 ± 6.82 ^a,c,d^	15.09 ± 3.22 ^a,b,c^	10.12 ± 3.78 ^a,b,c^	<0.001

COG, control group; BOG, bosentan group; ATG, atorvastatin group; and BO + ATG, bosentan + atorvastatin group. *p*, one-way ANOVA *p* value. Tukey test, significant differences in each intervention group (*p* < 0.05) compared to other groups based on post hoc one-way ANOVA analysis: ^a^ COG, ^b^ BOG, ^c^ ATG, and ^d^ BO + ATG.

## Data Availability

The raw data supporting the conclusions of this article will be made available by the authors on request.
